# *Tobacco mosaic virus* Movement Protein Enhances the Spread of RNA Silencing

**DOI:** 10.1371/journal.ppat.1000038

**Published:** 2008-04-04

**Authors:** Hannes Vogler, Myoung-Ok Kwon, Vy Dang, Adrian Sambade, Monika Fasler, Jamie Ashby, Manfred Heinlein

**Affiliations:** 1 Department of Plant Physiology, Botanical Institute, University of Basel, Basel, Switzerland; 2 Friedrich Miescher Institute for Biomedical Research, Basel, Switzerland; 3 Institut Biologie Moléculaire des Plantes, Laboratoire propre du CNRS (UPR 2357) conventionné avec l'Université Louis Pasteur (Strasbourg 1), Strasbourg, France; The Scripps Research Institute, United States of America

## Abstract

Eukaryotic cells restrain the activity of foreign genetic elements, including viruses, through RNA silencing. Although viruses encode suppressors of silencing to support their propagation, viruses may also exploit silencing to regulate host gene expression or to control the level of their accumulation and thus to reduce damage to the host. RNA silencing in plants propagates from cell to cell and systemically via a sequence-specific signal. Since the signal spreads between cells through plasmodesmata like the viruses themselves, virus-encoded plasmodesmata-manipulating movement proteins (MP) may have a central role in compatible virus:host interactions by suppressing or enhancing the spread of the signal. Here, we have addressed the propagation of GFP silencing in the presence and absence of MP and MP mutants. We show that the protein enhances the spread of silencing. Small RNA analysis indicates that MP does not enhance the silencing pathway but rather enhances the transport of the signal through plasmodesmata. The ability to enhance the spread of silencing is maintained by certain MP mutants that can move between cells but which have defects in subcellular localization and do not support the spread of viral RNA. Using MP expressing and non-expressing virus mutants with a disabled silencing suppressing function, we provide evidence indicating that viral MP contributes to anti-viral silencing during infection. Our results suggest a role of MP in controlling virus propagation in the infected host by supporting the spread of silencing signal. This activity of MP involves only a subset of its properties implicated in the spread of viral RNA.

## Author Summary

RNA silencing is a fundamental mechanism that, among other important tasks, controls the accumulation of viruses through the degradation of their RNA intermediates. Since viruses encode suppressors of RNA silencing it is assumed that RNA silencing has evolved as an antiviral defense response. Thus, the idea of an arms race between the virus and the host, which the virus has to win for a successful infection, is now widely accepted. Our results question this concept of an arms race by showing that a virus-encoded protein, the movement protein (MP) of *Tobacco mosaic virus* (TMV), supports the intercellular trafficking of the non-cell-autonomous silencing signal. A virus mutant with defects in the suppressor is shown to be more prone for silencing with MP than without MP indicating that MP supports antiviral silencing during infection. Previous studies have demonstrated that the expression of silencing suppressors leads to viral overaccumulation and the death of the plant. Therefore, we suggest that the ability of MP to support the spread of signal may contribute to the control of virus propagation in the infected host.

## Introduction

Recent research has revealed an elegant antiviral defense mechanism in plants, vertebrates, and invertebrates that works through sequence-specific degradation of RNA [1]–[8]. RNA silencing is triggered by double-stranded RNA (dsRNA) [9] and associated with the accumulation of short 21- to 24-nt RNAs (siRNAs) [10],[11] that are generated upon cleavage of dsRNA by dicer or dicer-like enzymes (DCL). Following their production, the siRNAs are incorporated into RNA-induced silencing complexes (RISC) that contain an ARGONAUTE (AGO) family protein (in plants, AGO1 [12]) and cleave cognate RNA molecules endolytically [13].

In the course of the silencing process in plants, a diffusible or transported RNA-based and sequence-specific signal is generated that moves through plasmodesmata and mediates the spread of RNA silencing throughout the organism [14]–[19]. Thus, once activated locally, silencing can spread between cells and into other plant organs, causing systemic inactivation of the target gene [16],[20]. Silencing signaling occurs in two phases [21]. The first phase results in silencing of cells up to 10–15 cells away from the cells, in which silencing was initially triggered. The second phase leading to systemic silencing depends on relay amplification of the signal in recipient cells and involves RDR6 (an RNA-dependent RNA polymerase) and SDE3 (a putative RNA helicase) [22]–[24].

It is now generally accepted that silencing in plants acts as a major antiviral defense mechanism [25],[26]. Here, silencing is triggered by viral dsRNA produced during replication. Subsequently, this dsRNA is cleaved by the dicer-like enzymes DCL2 and DCL4, and the resulting siRNA is used to program a RISC for the degradation of the cognate viral genome [27]. Viruses counteract this silencing by evasion, e.g. by minimizing production and exposure of dsRNA, as well as by suppression, i.e. through expression of proteins that interfere with the silencing pathway [25],[26]. On the other hand, silencing is enhanced by the production of the non-cell-autonomous silencing signal, which has been proposed to prime RISC in non-infected cells for degradation of the incoming virus [25]. Since expression of silencing suppressor leads to viral overaccumulation and soon to the death of the infected plant [28],[29], the spread of silencing signal may play an essential counter-balancing role in controlling the accumulation of the virus in newly infected cells. Interestingly, the silencing signal propagates between cells through plasmodesmata [17], just like the viruses themselves [30]. This suggests that the virus-encoded movement proteins (MP) may play an important role in regulating this counter-balancing relationship by restricting or enhancing the spread of the signal and, thus, in ensuring a successful virus:host interaction.

## Results

**Figure 1:**
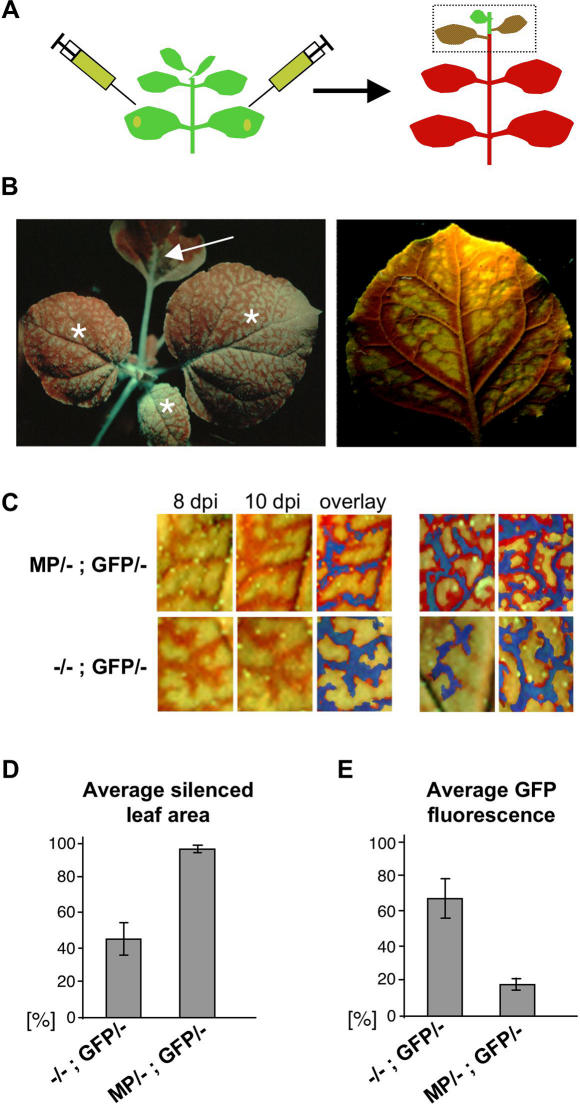
MP enhances the spread of GFP silencing in systemic leaves. (A) Systemic GFP silencing in *N. benthamiana* line 16c was induced by agroinfiltration of a GFP expressing construct into lower leaves. Silencing in upper, non-infiltrated leaves was analyzed. (B) A plant under UV illumination in which GFP silencing has spread from infiltrated leaf (arrow) into non-infiltrated, upper leaves (asterisks). An example of an upper leaf showing the pattern of GFP silencing spreading from class I–III veins into adjacent tissue is shown on the right. (C) Efficiency of cell-to-cell spread of GFP silencing during 36 h in segments of upper, non-infiltrated leaves that where heterozygous for GFP and MP (MP/-; GFP/-, top row) or heterozygous for GFP alone (-/-; GFP/-, second row). The first and second panels in each row show the silencing pattern at 8 dpi and 36 h later (10 dpi), respectively. The third panels show overlays of the first two panels. Blue false color represents the silenced area at 8 dpi (as shown in first panels) and red enhanced color indicates the increase of silenced areas after the 36 h incubation period (as shown in second panels). Panels four and five in each row show similar overlays made from different source images. At 10 dpi the area of newly silenced tissue (shown in red artificial color) in these plants was considerably greater in the presence than in the absence of MP. (D) and (E) Quantification of GFP silencing in upper leaves of 10 plants each of either MP-expressing plants (MP/-; GFP/-) and control plants (-/-; GFP/-). (D) Percentage of silenced leaf area as revealed by the number of green pixels (representing non-silenced area) and red pixels (representing silenced area) in digital leaf images. (E) Percentage of GFP fluorescence in leaf extracts (compared to GFP fluorescent control leaves = 100%).

To test whether viral MP may influence the spread of silencing signal, we investigated the spread of RNA silencing in the presence and absence of the prototypical MP, the 30 kDa MP of *Tobacco mosaic virus* (TMV). The capacity of this non-cell-autonomous protein [31] to facilitate the spread of the viral RNA has been associated with its ability to modify the size exclusion limit (SEL) of plasmodesmata [32],[33], to bind RNA [34], and to associate with endoplasmic reticulum (ER) and derived structures, as well as with microtubules [35]–[39]. To visualize the spread of RNA silencing, we used TMV-susceptible *Nicotiana benthamiana* plants that express green fluorescent protein (GFP) (line 16c) [40]. RNA silencing of GFP was induced by agroinfiltration of GFP sequences (Figure 1A) and visualized by the disappearance of GFP fluorescence (Figure 1B), as has been described [17]. Previous studies have established that the spread of GFP silencing is independent of the method for delivery of the inducing construct and not caused by recurrent transfection of cells by spreading *Agrobacterium* [17]. To test whether the cell-to-cell transport of the gene-silencing signal is influenced by a viral MP, the spread of GFP silencing was analyzed in heterozygous *N. benthamiana* F1 hybrids between the homozygous GFP-transgenic plant line 16c and plant line NB15 homozygous for the MP of TMV [41]. The MP-transgenic *N. benthamiana* plants complement the movement of MP-deficient virus, indicating that transgenic MP is expressed and functional [41],[42] (Figure S1).

**Figure 2:**
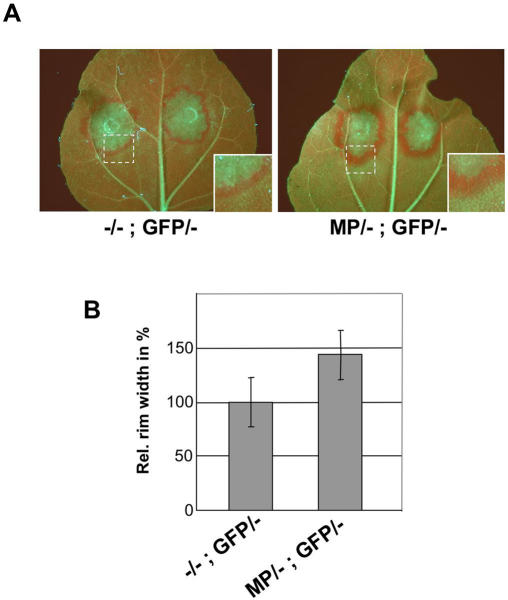
MP enhances cell-to-cell spread of local GFP silencing. (A) Examples showing the rim of GFP-silenced cells surrounding the agroinfiltrated leaf areas. Magnifications of the highlighted areas (dashed boxes) are shown at the lower right. (B) Relative average width of the silenced rim in plants expressing no MP (-/-; GFP/-) or MP (MP/-; GFP/-). The relative average widths of the silenced area around 20 agroinfiltrated patches each are shown. Error bars show the standard deviations. The mean value for the control infiltration (-/-; GFP/-) was set to 100%. The statistical significance of the MP effect was confirmed by ANOVA with a Tukey's HSD (*P*<0.01).

Following agroinfiltration of the plants with a GFP construct to induce silencing, it became obvious that the efficiency of the spread of GFP silencing, although variable to some extent, is overall increased in the presence of MP. To analyze the effect of MP on the spread of GFP silencing, the cell-to-cell progression of GFP silencing was examined over time in individual leaves (Figure 1C). The leaves were taken from plants undergoing systemic silencing at 8 and 10 days post infiltration (dp), in order to monitor the spread of GFP silencing from class I–III veins into adjacent tissue before the leaves became fully silenced. Upon close inspection of the leaves, it was evident that within two days the spread of silencing has progressed more efficiently in the heterozygous plants expressing MP (MP/-; GFP/-) than in control plants expressing no MP (-/-; GFP/-). We also investigated plants that were homozygous for MP and GFP (MP/MP; GFP/GFP) and found again that GFP-silencing progressed more efficiently in the MP-transgenic plants compared to MP non-transgenic control plants (-/-; GFP/GFP) (Figure S2). The effect of MP on the spread of silencing in the systemic leaves was also evident when 10 leaves each (leaves undergoing the spread of silencing, i.e. leaves in equal position above the infiltrated leaf) of MP-expressing plants (MP/-; GFP/-) and control plants (-/-; GFP/-) were compared with respect to their average GFP silenced area (Figure 1D) or with regard to their average amount of remaining GFP fluorescence (Figure 1E). The MP also enhanced the spread of local silencing in the agro-infiltrated leaves. As shown in Figures 2A and B, the rim of silenced tissue that develops around the patch of agro-infiltrated cells upon the initiation of local silencing [21] was considerably wider in the heterozygous MP-expressing 16c plants (MP/-; GFP/-) compared to 16c plants expressing no MP (-/-; GFP/-). In two independent experiments the width of red fluorescent silenced tissue was increased by 44% (Figure 2B) and 36% (not shown). In average, the presence of MP increased the number of locally silenced cell layers by about 5 cells from 10–15 to 15–20 cells.

**Figure 3:**
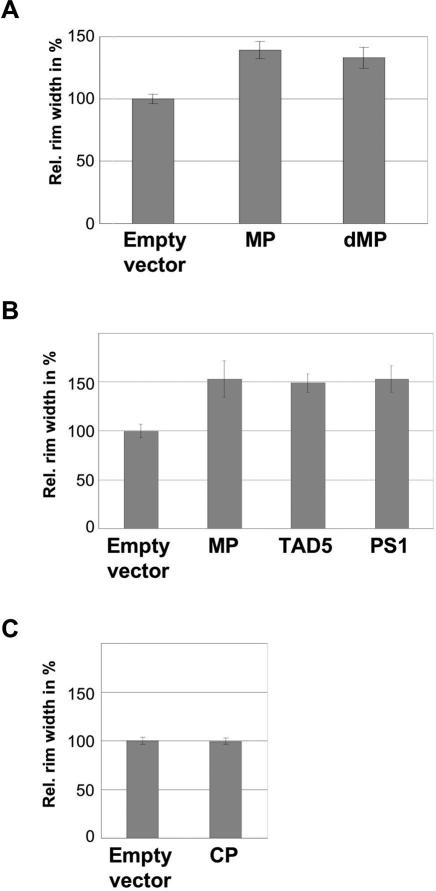
MP enhances cell-to-cell spread of local GFP silencing upon transient expression in agroinfiltrated leaves. (A) Transiently expressed MP enhances cell-to-cell spread of local GFP silencing. A positive effect on the spread of local silencing is also seen upon transient expression of dMP. The relative average widths of the silenced area around agroinfiltrated patches are shown. Error bars show the standard deviations. The value derived from the control infiltration (empty vector) was set to 100%. The statistical significance of the measured differences was confirmed by ANOVA with a Tukey's HSD test (Empty vector (*n* = 18) vs. MP (*n* = 36), *P*<0.01; Empty vector vs. dMP (*n* = 36), *P*<0.01). (B) The enhancing effect of transiently expressed MP on the short-distance cell-to-cell spread of GFP silencing is not affected by mutations that interfere with accumulation at microtubules (TAD5) or with accumulation at both microtubules and plasmodesmata (PS1). The relative average widths of the locally silenced areas surrounding agroinfiltrated patches are shown. All tested mutant MP variants enhance the short-distance spread of GFP silencing as wild type MP. Error bars show the standard deviations. The statistical significance of the measured differences was confirmed by ANOVA with a Tukey's HSD test (Empty vector (*n* = 48) vs. MP (*n* = 54), *P*<0.01; Empty vector vs. TAD5 (*n* = 42), *P*<0.01; Empty vector vs. PS1 (*n* = 48), *P*<0.01). (C) Expression of CP does not enhance the local spread of silencing. Relative widths of the leaf area surrounding agroinfiltrated patches are shown. Error bars show the standard deviations. ANOVA with a Tukey's HSD was performed (empty vector (*n* = 36) vs. CP (*n* = 36); *P* = 0.41).

**Figure 4:**
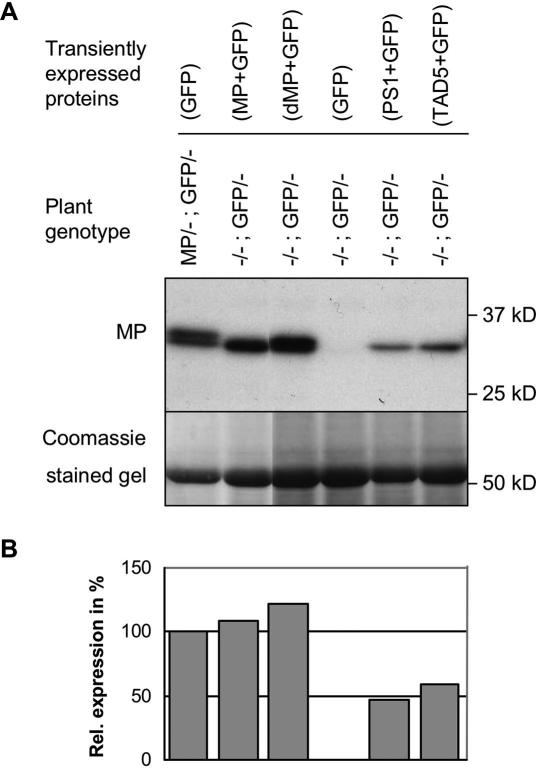
Level of MP expression in transgenic plants and upon agroinfiltration. (A) Westernblot analysis of protein extracts produced at 3 dpi. MP is detected with specific antibody. The plant genotype and the proteins expressed by transient expression are stated above each lane. Coomassie staining of the same membrane reveals the amount of Rubisco, which was used for normalization in (B). Transiently expressed MP and MP expressed from the transgene accumulate to similar levels in the cells. PS1 and TAD5 expression levels are lower. (B) Quantification of MP levels based on the Westernblot shown in (A). The differences in gel loading as revealed by Coomassie staining (A, lower panel) were used for normalization. The normalized level of MP produced from the transgene was set to 100.

Similar to transgenic plants, MP increased the spread of silencing also under conditions of transient expression (Figure 3A). The transiently expressed MP is functionally active as demonstrated by the spread of MP-deficient virus in the agroinfiltrated leaves (Figure S3). In order to test whether the ability of MP to increase the spread of silencing reflects functions of the protein associated with the spread of viral RNA, we included MP mutants in our assay. The first mutant tested was defective MP (dMP; [43]), which carries a deletion of three amino acids (Δaa3–5) and fails to complement for the movement of a MP-deficient virus in transgenic tobacco plants. This functional defect has been correlated with the observation that this protein has reduced capacity to accumulate in plasmodesmata and causes only a partial increase in plasmodesmal SEL [44]. In addition, unlike wild type MP, this mutants shows extensive accumulation on microtubules when expressed during infection in tobacco protoplasts or transiently in *N. benthamiana* leaves ([43]; Figure S4). Surprisingly, when tested in our transient assay, dMP produced a similar effect on the spread of silencing as wild type MP (Figure 3A). The ability of MP to increase the spread of silencing thus relies on MP functions that are insufficient for the spread of viral RNA. Indeed, further tests revealed that also other MP mutants known to be defective in viral RNA transport still have the ability to enhance the spread of silencing under conditions of transient expression (Figure 3B). Like dMP, also these mutants differ from MP with regard to subcellular targeting and thus provide additional clues as to which interactions of MP with subcellular components may be dispensable for its ability to facilitate the spread of silencing. One mutant is TAD5, which like dMP carries a three amino acid deletion mutation (aaΔ49–51). TAD5 localizes to ER and ER-derived structures but, unlike wild type MP and dMP, does not associate with microtubules. However, during infection in complementing MP-transgenic plants, the protein still localizes to plasmodesmata [45]. Another tested mutant is MP^P81S^ (referred here to as “PS1”), which carries a previously characterized inactivating amino acid replacement mutation (P81S) [46],[47]. In contrast to TAD5, PS1 fails to localize to any subcellular structure in protoplasts [47]. Although this mutant protein still tends to accumulate in plasmodesmata when expressed during virus infection in complementing MP-transgenic plants, it fails to accumulate in plasmodesmata when expressed alone in agroinfiltrated leaves (Figure S4). Given that even PS1 still facilitates the spread of silencing, it appears that neither association with microtubules or ER, nor accumulation in plasmodesmata plays a role in the ability of the MP to enhance the spread of silencing under transient expression conditions. Importantly, expression of the TMV coat protein (CP) had no effect on the spread of silencing (Figure 3C). To test whether the ability of MP and MP mutants to facilitate the spread of silencing in our transient assay might involve an artefact due to overexpression, an immunoblot analysis was performed. However, irrespective whether samples were taken at 3 days post agroinfiltration (3 dpi) (Figure 4) or at 5 dpi (data not shown), we found that the levels of transiently expressed MP and dMP were comparable to the level of MP in MP-transgenic plants. PS1 and TAD5 proteins even accumulated to lower levels. Collectively, these results indicate that MP exerts a specific promoting effect on the spread of silencing. However, the mechanism relies on MP functions other than microtubule association and anchorage in plasmodesmata.

**Figure 5:**
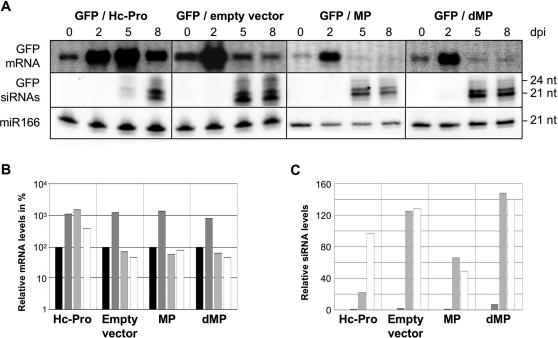
Expression of MP does not interfere with the silencing pathway. (A), The levels of GFP mRNA, GFP siRNA, and miR166 (loading control) at different time points (0, 2, 5, and 8 dpi) in agroinfiltrated tissues. The patterns of GFP mRNA and siRNA in MP- or dMP-expressing tissue are similar as in tissues expressing empty vector. GFP mRNA levels peaks at 2 dpi due to the expression of GFP from the silencing inducer construct. Between 2 and 5 dpi a strong decrease below the original levels (0 dpi) of GFP mRNA is observed. This indicates that in addition to the GFP expressed from the silencing inducer construct the GFP expressed from the transgene is silenced. Hc-Pro, a known silencing suppressor, prevents degradation of GFP mRNA, which peaks at 5 dpi. GFP siRNAs appear at 5 dpi. Expression of Hc-Pro delays the accumulation of siRNAs. (B), Quantification of mRNA levels. mRNA patterns reveal no effects of both MP and dMP on GFP silencing. The values for 0 dpi were set to 100%. Black bars: 0 dpi; dark grey bars: 2 dpi; light gray bars: 5 dpi; white bars: 8 dpi. (C) Quantification of siRNA levels. In the presence of MP, the GFP siRNA levels are reduced by about 50% compared to the empty vector control, whereas dMP causes a slight increase. The values for 0 dpi were set to 0. Dark grey bars: 2 dpi; light gray bars: 5 dpi; white bars: 8 dpi.

The extent of non-cell-autonomous silencing has been correlated with the amount of 21 nt siRNAs [48]. To test whether the mechanism by which MP increases the extent of cell-to-cell movement of GFP silencing involves an effect of MP on the level of GFP siRNAs, we analysed the levels of GFP mRNA and siRNAs by northernblot hybridization at various time points in patches co-agroinfiltrated with constructs that express MP and GFP (Figure 5). In control experiments, we also analysed leaves co-infiltrated with constructs expressing GFP and Hc-Pro, a well characterized potyviral silencing suppressor that sequesters siRNA and therefore inhibits the availability of siRNA for RISC assembly [49]. As shown in Figure 5, expression of GFP together with empty control vector led to an increase in GFP mRNA at 2 dpi. Subsequently, at 5 dpi and 8 dpi and concomitant with the appearance of GFP siRNAs, the level of GFP mRNA decreased. In contrast to empty vector, expression of Hc-Pro resulted in the stabilization of GFP mRNA beyond 2 dpi and a corresponding delay in the appearance of accumulating GFP-specific siRNAs. In contrast, replacement of empty vector expression with expression of either MP or dMP had no significant effect on the GFP mRNA accumulation pattern. Similar to the control experiment, a strong increase in GFP mRNA level at 2 dpi was followed by a decrease at 5 dpi and 8 dpi. Thus, unlike Hc-Pro expression, MP expression does not lead to a stabilization of GFP mRNA. Quantification showed that siRNA levels are slightly decreased rather than increased in the presence of MP compared to the empty vector control (Figure 5C). However, this feature seems to be unrelated to the ability of MP to facilitate the spread of silencing, since dMP, which also facilitates the spread of silencing, did not cause significant changes in the level of siRNAs (Figure 5C). Taken together, based on these observations and verified by a replicate experiment (not shown), we conclude that unlike Hc-Pro, MP does not significantly affect the silencing pathway; a positive correlation between the amount of 21 nt siRNAs and the spread of the silencing signal was not observed. Similar results were obtained for tissues expressing TAD5 or PS1 (Figure S5A) and also the expression of the viral coat protein (CP) had no effect on GFP mRNA and siRNA levels (Figure S5B). Therefore, we conclude that MP does not enhance the spread of local silencing in infiltrated tissues by increasing the level of siRNAs.

**Figure 6:**
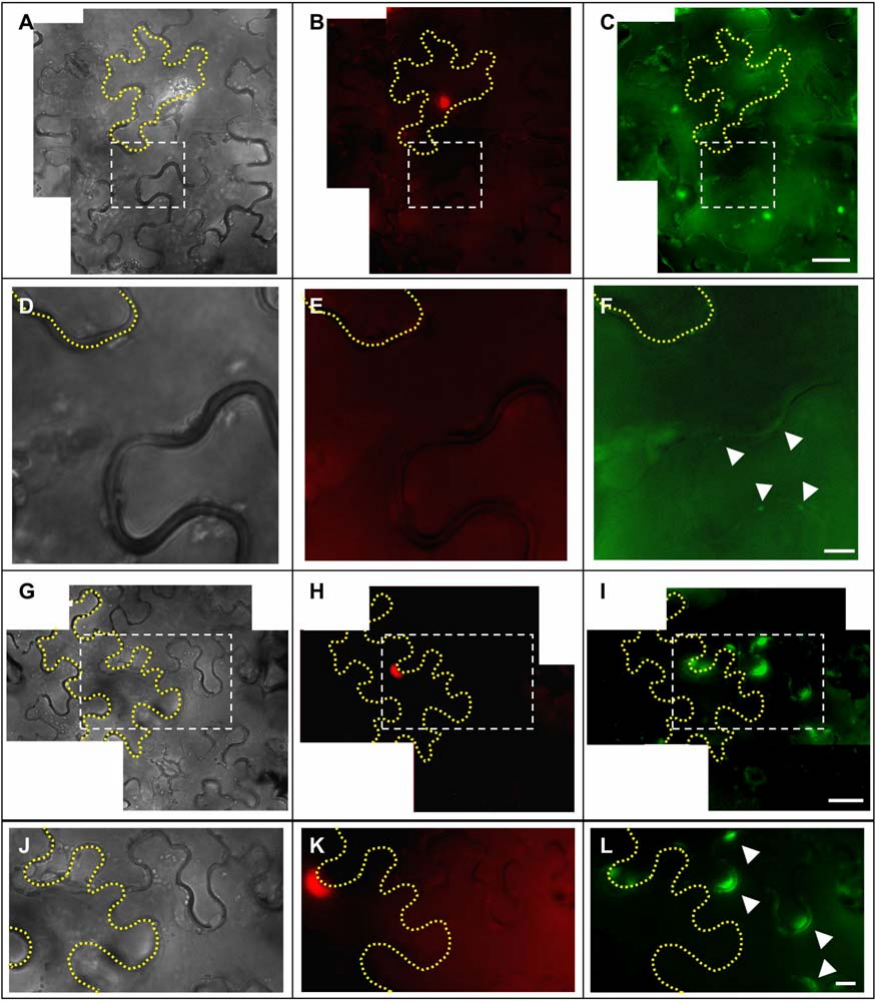
PS1:GFP moves cell-to-cell. Single epidermal cells from *N. benthamiana* leaves were infiltrated with agrobacteria co-transformed with plasmids for specific co-expression in the same cells of the red fluorescent cell-autonomous nuclear marker RMS2 together with either MP:GFP (A to F) or PS1:GFP (G to L). (Left panels: DIC; middle panels: RFP channel; right panels: GFP channel). (A–F), Movement of MP:GFP. Movement is indicated by the presence of punctate MP:GFP fluorescence (plasmodesmata) in the cell wall of a cell distant to the transfected cell (arrowheads). (A) DIC image of epidermis. The transfected cell is indicated by a yellow, dotted line. Area delimited by dashed line is magnified in (D). (B) RFP channel image showing transfected cell as indicated by presence of the red fluorescent nuclear protein RMS2. Area delimited by dashed line is magnified in (E). (C) GFP channel image. Area delimited by dashed line is magnified in (F), indicating the presence of MP:GFP at plasmodesmata of non-transfected cells (arrowheads). (G–L) Movement of PS1. Movement is indicated by the presence of diffuse PS1:GFP fluorescence in the cell adjacent to the transfected cell (arrowheads). (G) DIC image of epidermis. The transfected cell is indicated by a yellow, dotted line. Area delimited by dashed line is magnified in (J). (H) RFP channel image showing transfected cell as indicated by presence of the red fluorescent marker RMS2. Area delimited by dashed line is magnified in (K). (I) Green channel image showing presence of PS1:GFP. Area delimited by dashed line is magnified in (L) indicating the presence of PS1:GFP in a non-transfected cell (arrowheads). Size bars: C, 50 µm; F, 10 µm; I, 50 µm; L, 10 µm.

Given that a significant modification of the silencing pathway seems not to play a role, it appears likely that the MP enhances the spread of silencing by its ability to modify the SEL of plasmodesmata and to move between cells [31]. Moreover, given that the MP has sequence non-specific nucleic acid binding activity [34] the protein may interact with the signal to enhance its transport. If this model were correct, a MP mutant such as PS1, which has retained nucleic acid binding activity [47] but does not accumulate on microtubules nor in plasmodesmata, and is also deficient in TMV RNA transport, should have preserved the ability to increase plasmodesmal SEL and to move between cells in order to enhance the spread of silencing. To test whether PS1 has retained the ability to move between cells, we transiently expressed PS1:GFP in single epidermal cells of *N. benthamiana* leaves and investigated the occurrence of GFP signals in adjacent non-transfected cells by fluorescence microscopy. To visualize the transfected cells and to confirm that the spread of GFP fluorescence is due to the spread of protein and not due to the spread of the transfecting agrobacteria, the cells were infiltrated with agrobacteria co-transformed with two plasmids, one plasmid encoding PS1:GFP and another plasmid encoding a cell-autonomous, red fluorescent protein (RFP)–tagged nuclear-targeted protein, to which we refer as RMS2. To ensure that the transfected cells are surrounded by non-transfected cells, the agrobacteria harboring both the PS1:GFP- and RMS2-encoding plasmids were highly diluted before tissue infiltration (Figure S6). As demonstrated in Figure 6 and Figure S6, in control experiments in which functional MP:GFP was expressed, MP:GFP fluorescence was detected at 6 dpi in cells adjacent to the originally transfected cell (Figure 6, A–F), confirming the ability of MP:GFP to spread cell-to-cell [31]. Interestingly, in four independent experiments, we found that, although PS1:GFP unlike MP:GFP does not accumulate in plasmodesmata, in 80% of the cases the protein was nevertheless detected as a diffuse, cell wall-proximal fluorescence in cells adjacent to the transfected cells (Figure 6, G–L). Similar observations were obtained when using a microparticle bombardment assay as described [50]. However, in this case, we were not able to reliably distinguish the diffuse PS1:GFP fluorescence from the diffuse autofluorescence exhibited by the mechanically damaged cells. Nevertheless, the results obtained by our agroinfiltration assay indicate that PS1:GFP has retained the capacity for intercellular movement and that the accumulation of the protein on microtubules or in plasmodesmata is not required for this activity. Since microtubule association and accumulation in plasmodesmata have been associated with the function of MP in viral RNA movement, it appears that MP potentiates silencing signal trafficking by a mechanism less complex than that involved in the trafficking of viral RNA and that the effect may simply be caused by the capacity of the protein to modify plasmodesmal SEL and/or to bind nucleic acids and to move between cells.

**Figure 7:**
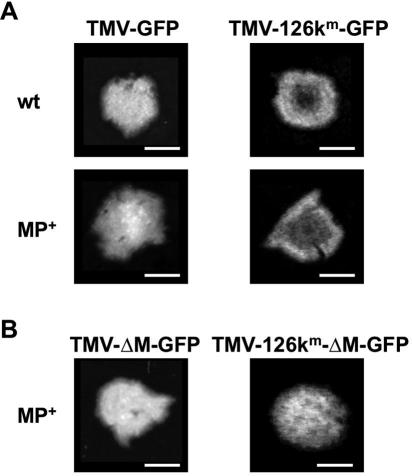
MP enhances silencing during infection. Green fluorescent infection sites of GFP-expressing TMV-derivatives in inoculated leaves of wild type and MP-transgenic (MP+) *N. benthamiana* plants. (A) Anti-viral silencing is exposed in infection sites caused by silencing suppressor-defective virus TMV-126k^m^-GFP [52] as seen by the disappearance of GFP fluorescence in the center of the infection sites. Scale bars: 5 mm. (B) Deletion of the MP gene from the virus (ΔM) abolishes induced silencing. In contrast to infection sites of TMV-126k^m^-GFP (A), infection sites of TMV-126k^m^-ΔM-GFP do not show any silencing. Scale bars: 5 mm.

To determine whether the ability of MP to potentiate silencing signaling plays a role during virus infection, *N. benthamiana* plants were infected with MP-bearing and MP-deficient TMV constructs that express GFP in place of coat protein. In order to expose the effect of MP on anti-viral silencing, we used constructs carrying an amino acid exchange mutation in the 126k replicase, which reduces the silencing suppressing activity of this protein [51],[52]. As a result of the mutation, induced silencing of the virus is no longer suppressed as is evident by a reduction of GFP fluorescence in the center of the infection site ([51],[52]; Figure 7A and Table 1). Since infection requires MP, it was necessary to use MP-transgenic plants for complementation of the MP-deficient constructs. As shown in Figure 7A and Table 1, the MP expressed from the transgene had no obvious influence on the initiation of silencing. Importantly, deletion of MP from the suppressor-deficient virus led to the loss of silencing in the center of the infection site (Figure 7B and Table 1) suggesting that viral MP contributes to silencing initiation and/or maintenance in infected cells. Time course observations revealed that the infection sites of MP-deficient constructs enlarged with the same efficiency in MP-transgenic plants as infection sites of MP-encoding viruses (Figure S7). Moreover, no silencing in the center of infection sites occurred within the investigated time periods (20 days post infection). Apparently, the 126k replicase protein and the viral MP may act in opposing, counter-balancing ways in controlling the level of anti-viral silencing and thus virus accumulation in infected cells.

**Table 1:** Frequency of centrally silenced infection sites.

	TMV-GFP	TMV-126k^m^-GFP	TMV-ΔM-GFP	TMV-126k^m^-ΔM-GFP
Wild type	0 (57)	133 (191)	n. a.	n. a.
MP^+^	0 (85)	71 (107)	0 (11)	0 (163)

Frequency of infection sites showing central silencing. The total number of infection sites analysed is shown in brackets. n. a.: not applicable.

It seems remarkable that transgenic MP complements viral MP deficiency for virus movement but not for virus silencing, at least not to any visible level. The reason for this finding is yet unknown. It may be conceivable that the MP RNA sequence deleted from the target virus is required for silencing triggering and that, therefore, the complementation by transgenic MP is without effect. However, a role of the MP protein rather than a role of MP RNA is likely given that MP expressed outside the virus context is able to facilitate the spread of post-transcriptional silencing of a different gene (GFP) *in trans*. Moreover, virus silencing usually involves structured RNA regions distributed along the whole viral genomic sequence [53],[54], which makes the possibility of the MP RNA sequence being essential for viral silencing seem rather unlikely. The obvious inability of transgenic MP to support antiviral silencing may be caused by the amount or specific location of MP produced from the transgene. Indeed, while MP produced from the virus accumulates at various sites in the cell [38], transgenic MP accumulates only in plasmodesmata if expressed in tobacco [55] or in other species, such as *N. benthamiana* (E. Boutant, C. Ritzenthaler, and M. Heinlein, data not shown) or Arabidopsis (A. Sambade and M. Heinlein, data not shown). Thus, while the amount and localization of MP encoded by the nuclear gene suffices to support the spread of single virus genomes and the spreading silencing of a nucleus-encoded transcript, the amount of MP and its accumulation exclusively at plasmodesmata may be insufficient to effectively support the degradation of the replicating virus genome.

## Discussion

The finding that MP facilitates the spread of silencing may indeed play an important role in compatible TMV:host interactions. Like other viruses, TMV is an obligate parasite, which must preserve the integrity of the host. Overaccumulation of a virus is detrimental to the host as shown by the fact that plants expressing silencing suppressors are hypersusceptible and often simply killed by the invading virus [28],[29]. Given this example, it appears likely that viruses like TMV that are able to suppress the host silencing defense response must also have mechanisms to avoid their own overaccumulation. Recent studies indicate that one way to protect the host is by exploitation of RNA silencing for self-attenuation. For example, subviral RNA species produced by many RNA viruses can act as strong inducers of anti-viral RNA silencing and thus play an important role in virus-host interactions (reviewed in: [56]). A strategy in animal DNA viruses is the production of virus-encoded miRNAs that target the viral genome itself [57],[58]. Another striking example of virus attenuation might be “recovery”, a silencing phenomenon where highly symptomatic plants suddenly show massive reduction of virus accumulation in newly grown tissue, although elimination is never complete [59]. It has been proposed that by this way, meristem infecting viruses could ensure pollen-transmission via healthy, flowering plants [25]. The ability of TMV MP to promote the spread of silencing may be another example of a self-attenuation mechanism. By allowing silencing to spread efficiently, the virus could promote anti-viral silencing in newly infected cells, thus controlling its accumulation. However, the role of MP in supporting the spread of silencing is likely limited to early stages of infection, since at later stages of infection, i.e. when infection has further advanced into newly infected cells and cells originally at the leading front are now in the center of the infection site, the expression of MP has decreased [38] and MP no longer increases plasmodesmal SEL [60]. Limiting the movement and silencing functions of MP to the leading front of infection appears logical as infected cells undergo transitions from early phases dedicated to virus movement to later phases rather dedicated to virus reproduction. The silencing promoting function of MP expressed at the front of infection may have no detrimental effects on the spread of the virus since the spread of infection requires only few virus genomes [61]. However, as the cells in which MP supported the establishment of silencing undergo later stages, a continued expression of MP and thus of a silencing promoting function would likely exert an inhibotory effect on viral reproduction. Thus, by limiting MP expression to the front of infection [38], by expressing a silencing suppressor [52], and by producing viral coat protein (CP) only during later stages of infection [62]–[64], the virus may be equipped with highly coordinated and interactive means to protect the host through restricting high levels of viral RNA accumulation to cells in which the RNA becomes encapsidated.

Given that MP has no significant effect on the accumulation of siRNAs and, thus, on the silencing pathway, it appears likely that MP enhances the spread of silencing by supporting the spread of the RNA-based silencing signal. The MP of TMV may not be the first example of a viral MP that promotes the spread of the silencing signal. Another example may be the 25 kDa “triple-gene-block” MP (TGBp1) of *White clover mosaic virus* (WClMV) which upon expression in transgenic *N. benthamiana* allowed the spread of silencing signal and viral RNA into meristem tissue, that normally remains free of these RNA molecules [65]. However, the authors of this finding did not discuss the potential importance of their finding with regard to viral self-attenuation.

Our results are insufficient to fully determine the mechanism by which the MP may support the spread of signal. However, the mechanism appears to be less complex than the mechanism involved in the transport of viral RNA, since several mutant MPs that are non-functional in virus transport are still capable of enhancing silencing spread. Specific subcellular localization does not seem to be required as opposed to the transport of viral RNA [36], [37], [42]–[45],[47],[66]. However, the mechanism may rely on the ability of MP to modify the SEL of plasmodesmata and to spread between cells [31],[33] as well as on the capacity to interact with nucleic acids in a sequence non-specific manner [34]. For example, we have shown here that the mutant MP protein PS1, which does not facilitate viral RNA transport and fails to accumulate in plasmodesmata and on microtubules [47], still facilitates the spread of local silencing. On the other hand, this mutant protein has retained the ability to bind nucleic acids [47] and the capacity to move between cells. Preliminary experiments indicate that PS1 has the ability to increase the SEL of plasmodesmata and to allow the spread of large 10 kDa dextran molecules (F. Kragler, personal communication). Thus, whether MP binds and co-transport silencing signal itself or whether it may rather allow a different silencing signal-containing complex to move through modified plasmodesmata remains to be investigated. The ability to facilitate the spread of silencing is also demonstrated for other MP mutants, such as dMP and TAD5. Likely, also these mutants have retained RNA binding activity and the ability to spread between cells, since the small mutations carried by PS1, dMP, and TAD5 are all located in the N-terminal domain of MP and thus are distant to the more centrally and C-terminally located domains in MP that need to be deleted to impede RNA binding [67] or interaction with plasmodesmata [31]. The conclusion that the MP facilitates the spread of silencing and that this activity involves the ability of the protein to bind RNA, to modify the plasmodesmal SEL, and to move between cells is consistent with the recent notion that the degree of silencing signal spread positively correlates with the permeability of plasmodesmata [68] as well as with the finding that silencing signals in plants may travel as ribonucleoprotein complexes [69]. The potential formation of a complex between the silencing signal and MP may be explored as a possibility to achieve the characterization of the yet elusive identity of the signal.

Collectively, the observations indicate that TMV and potentially other plant viruses encode one or more proteins that interact with the silencing machinery of the host in diverse ways. The finding that TMV may not only suppress but also exploit small RNA pathways in a positive sense argues against the concept that successful infections primarily depend on the ability of a virus to overcome silencing and thus to win an arms race with the host [27],[70]. Rather, it appears that a successful infection, which can indeed only occur if neither the virus nor the host is destroyed, relies on the ability of the virus to tightly control its accumulation through application of a highly adapted silencing suppression and exploitation regime.

## Materials and Methods

### Plant material

Line 16c of *Nicotiana benthamiana* carrying an mGFP5-ER reporter gene under the control of a *Cauliflower mosaic virus* 35S promoter was generated by Ruiz et al. [40]. Transgenic *N. benthamiana* line NB15 expressing wild-type TMV MP [41] was crossed with plants of line 16c to obtain progeny plants heterozygous for both TMV MP and GFP (MP/-; GFP/-). Heterozygous 16c plants were obtained from a backcross of line 16c into wild type *N. benthamiana*. MP/-; GFP/- plants were self-fertilized and progeny homozygous for both transgenes (MP/MP; GFP/GFP) was identified by PCR. Heterozygous plants were used for the experiments except where mentioned otherwise.

Wild type and transgenic plants were grown from seeds and maintained in approximately 70% humidity at 23°C with a 16-hour photoperiod. 3 to 4 weeks old plants were used for infiltration assays and inoculation experiments.

### Constructs

The plasmid encoding TMV-GFP is identical to plasmid pTMV-ΔC-GFP encoding TMV-ΔC-GFP, which has been described [35]. The plasmid encoding infectious cDNA for TMV-ΔM-GFP was created by replacing the NcoI-KpnI fragment of pU3/12ΔM-RV, thus the 3′ end of TMV-ΔM-RV [71], with the NcoI-KpnI fragment of pTMV-ΔC-GFP. Thus, TMV-ΔM-GFP expresses the GFP from the CP subgenomic promoter just like TMV-GFP but encodes a dysfunctional, truncated MP lacking the N-terminal 162 amino acids (MP consists of 268 amino acids). Silencing of GFP in GFP-transgenic plants was induced by infiltration of leaves with *Agrobacterium tumefaciens* strain GV3101 transformed with p35S:GFP, a pBIN construct that expresses GFP under the control of the *Cauliflower mosaic virus* (CaMV) 35S promoter. For expression of MP by agroinfiltration, the respective DNA sequence was amplified by PCR with primers HV0407 (5′GTGGATCCATATGTATAGATGGCTCTAGTTG3′) and HV0408 (5′CGAGTACTAGTTTAAAACGAATCCGATTC3′), using pU3-12/4 encoding the wild type TMV sequence [72] as template. The amplified DNA was digested with NdeI and SpeI and cloned into the corresponding sites of pG35Somega, a derivative of pGREENII0029 [73] into which an expression cassette under the control of the CaMV 35S promoter and the TMV omega leader (a translation enhancer) was inserted. Plasmid pG35Somega expressing the MP was renamed pGMP. The same procedure was used to create plasmids encoding mutant MP proteins dMP (pGNT1), PS1 (PGPS1), and TAD5 (pGTAD5). dMP was cloned by PCR using primers Vpf3 (5′GTGGATCCATATGTATAGATGGCTAAAGGAAAAGTG3′) and HV0408, and pU3-12/4 as template. pGTAD5 was created by amplification of a fragment from the cDNA encoding TAD5 virus [45] using primers HV0407 and HV0408. The fragment was then digested with NdeI and PspOMI and used for exchange of the equivalent fragment of pGMP, leading to pGTAD5. pGPS1 was created following PCR amplification of a DNA fragment encompassing the P81S mutation, using primers HV0407 and GFP-597 (-) (5′GGACAGGTAATGGTTGTCTG3′), and Tf5-PS1:GFP [47] as template. Subsequently the fragment was digested with NdeI and PspOMI and used for replacement of the NdeI-PspOMI fragment of pGMP.

Similarly, we created pGCP and pGRFP. The CP DNA was amplified from pU3-12/4 using primers HV0409 (5′CTGGATCCATATGTATGTCTTACAGTATCAC3′) and HV0410 (5′CAACTAGTCATCTTGACTACCTCAAGTTG3′), whereas the RFP cDNA was amplified from pTf5-MP:RFP [74] using primers RFP585_f (5′GAGGATCCATATGAGTTCATGAGGTTTAAGG3′) and RFP585_r (5′GGACTACTAGTTTAAAGGAACAGATGGTG3′). After digestion with NdeI and SpeI, both amplification products were cloned into NdeI/SpeI-digested pG35Somega. pG35Somega was used as ‘empty vector’, where mentioned in the text and figures. Plasmids pGMP:GFP, pGPS1:GFP and pGNT1:GFP are binary vectors encoding MP:GFP, PS1:GFP, and dMP:GFP, respectively. These plasmids were created by amplification of the respective cDNA sequences, including the Stop codon, by using Tf5-NX2:GFP, Tf5-PS1:GFP and Tf5-NT1:GFP [47] as template and primers 5′TGGCTCTAGTTGTTAAAGG3′ (5′ phosphorylated) and 5′CAATTATTTAGCGG3′ for MP:GFP and PS1:GFP, and primers 5′TGGCTAAAGGAAAAGTG3′ and 5′CAATTATTTAGCGG3′, for dMP:GFP. Subsequently, the fragments were digested with SpeI and cloned into NdeI (filled in)/SpeI digested pG35Somega. For agroinfiltration experiments, the plasmids were transformed into *Agrobacterium tumefaciens* (GV3101) containing pSoup [73].

pBSGFP5 was constructed by introducing the AvrII/XhoI fragment of Tf5-NSPAX into the XhoI/SpeI digested pBluescript SK+. Tf5-NSPAX is Tf5-NX2:GFP containing additional SalI, PauI (BssHII), and AvrII sites on an in frame linker sequence separating the MP and the GFP5 open reading frame (ORF) (Boyko V. and Heinlein M., unpublished). Gateway cloning was used to create pRMS2, a binary plasmid encoding the bacteriophage MS2 coat protein [75] N-terminally fused to red fluorescent protein (RFP) and the SV40 nuclear localization sequence (NLS). The recombination reaction was performed by using donor vector pDONR/Zeo-NLS:MS2-CP and destination vector pB7WGR2 (VIB, Ghent). For creating the donor vector, the NLS-MS2CP:GFP ORF was amplified from plasmid pG14-MS2-GFP (kindly provided by Robert Singer, Albert Einstein College of Medicine, New York) and subcloned into pGEM easy (Invitrogen) to create pGEM-NLS-MS2CP:GFP. This vector was then used as a template to create a PCR product containing the ORF for NLS-MS2CP flanked by *att* recombination sites. pDONR/Zeo-NLS:MS2-CP finally resulted from a BP recombination reaction between the PCR product and pDONR^TM^/Zeo (Invitrogen). Plasmid pBIN-P19 was kindly provided by O. Voinnet (IBMP, Strasbourg, France).

### Virus inoculation and agroinfiltration

*N. benthamiana* plants were mechanically inoculated in the presence of carborundum with infectious transcripts made from in vitro reactions using the MEGAscript®T7 Kit (Ambion).

For agroinfiltration experiments to investigate the spread of silencing, we followed the method of Voinnet and colleagues [76]. Bacteria were grown in 50 ml LB medium containing 50 µg/ml kanamycin and 20 µM acetosyringone at 28°C for 24 to 36 h. Cells were harvested by centrifugation and resuspended in 10 mM MgCl_2_, 10 mM MES and 100 µM acetosyringone to reach an OD_595_ of 0.5. Before infiltration the cells were incubated for 3 h to overnight at room temperature. Cells containing the silencing inducer construct p35S:GFP were mixed in a ratio of 1∶1 with cells harboring the test constructs. Final agrobacterium concentration was kept constant at OD_595_ = 0.5 throughout all experiments except for cell-to-cell transport experiments (Figure 6). The mixtures were co-infiltrated into leaves 4 to 6 of 3 to 4 week old plants.

Agrobacteria (GV3101) used in cell-to-cell transport experiments (Figure 6) were co-transformed with either pGMP:GFP or pGPS1:GFP together with RMS2 and pSOUP. These cells were co-infiltrated at an OD_595_ = 0.001 with agrobacteria carrying a pBIN-P19 construct (OD_595_ = 0.04). The localization of MP and PS1 was analyzed at 6–8 dpi.

### Imaging

GFP expressing tissues were illuminated with a BLAK RAY B-100AP UV-lamp (UVP Inc., Upland, Ca.) and images were captured using a Canon EOS-300D digital camera equipped with Canon EF-S 18–55 mm objective lens and WRATTEN gelatine filters (Kodak). Images were imported into Adobe Photoshop 6.0.1 software (Adobe Systems, Inc.) for further analysis.

The width of silenced tissue surrounding agroinfiltrated patches was measured using ImageJ software. Average and standard deviation for each treatment were calculated and a t-Test was performed to determine if differences between control and protein expressing samples were significant. Values of control experiments were set to 100%.

Fluorescence microscopy was usually performed with a Nikon Eclipse 80i equipped with CFI Plan Apochromat objectives (Nikon Corp., Tokyo, Japan). Filter set XF100 (Omega Optical Inc., Brattleboro, Vt.) was used for visualization of GFP. Leaf tissues were analyzed under 60× oil immersion objective lens and images were acquired and processed using an ORCA-ER 1394 digital camera (Hamamatsu Photonics, Hamamatsu City, Japan) and Openlab version 3.1.7 software (Improvision, Coventry, England). Images were prepared for printing using Adobe Photoshop version 6.0.1 (Adobe Systems Inc.).

To examine the cell-non-autonomous spread of MP:GFP and PS1:GFP a Nikon TE2000 inverted microscope was used. Leaf tissue was analyzed under a 40× CFI Plan Apochromat oil immersion lens and specific filtersets were used to visualize GFP and RFP fluorescence. Images were acquired with a Coolsnap HQ camera (Roper Scientific) and Metamorph 6.1 software (Universal Imaging).

### Determination of GFP fluorescence in extracts

For quantification of GFP fluorescence in extracts, leaves were ground in liquid nitrogen and extracted in protein extraction buffer (62.5 mM Tris-HCl, pH 6.8; 1% [w/v] SDS, 20% [v/v] glycerol). Samples were cleared by centrifugation and the protein concentrations were normalized before fluorimeter measurements (excitation 485 nm, emission 538 nm).

### RNA analysis

Harvested leaves were homogenized in liquid nitrogen and aliquots of 500 mg plant tissue were treated with 5 ml TRIzol reagent (Invitrogen) according to manufacturer's instructions in order to extract total RNA.

5 µg of glyoxylated total RNA were separated on a 1.2% TAE agarose gel and transferred to a Hybond N+ membrane (Amersham Biosciences). After UV crosslinking membranes were pre-hybridized with DIG Easy Hyb (Roche) for 30 min at 68°C. DIG-labelled GFP probes were made by PCR amplification in the presence of DIG-labelled dNTPs using pBSGFP5 as template and primers HV0431 (5′CAACTACAACTCCCACAACG3′) and GFP-597(-). Hybridization with DIG-labelled probe and washing of the membranes were performed according to the manufacturer's instructions at 68°C (Roche).

For the analysis of small RNA species 30 µg of total RNA were separated on a 15% polyacrylamide 8M Urea gel in 1× TBE. Small RNAs were transferred to a Hybond N+ membrane (Amersham Biosciences) by electroblotting in 1× TBE buffer for 14 to 16 h. After UV-crosslinking, membranes were hybridized for 14 to 16 h at 35°C in ULTRAhyb®-Oligo buffer (Ambion) in the presence of oligo probes end-labelled with ^32^P by T4 polynucleotide kinase (Roche) and purified through MicroSpin™ G-25 columns (Amersham Biosciences). Membranes were washed 2× 30 minutes at 35°C with 2× SSC, 0.5% SDS. Signals were detected after 4 h to 3 d exposure to phosphor screens using a Molecular Imager (BioRad).

### Protein analysis

Agroinfiltrated leaf tissue was ground in liquid nitrogen and proteins were extracted from 20 mg of tissue powder in 2× SDS-PAGE loading buffer (90 mM Tris-Cl, pH 6.8, 20% glycerol, 2% SDS, 0.02% bromophenol blue, 0.1 M DTT) by boiling the samples for 5 minutes. After size-fractionation on a 12% polyacrylamide gel, proteins were electro-blotted onto an Immun-Blot^TM^ PVDF membrane (BioRad) in 25 mM Tris, 197 mM glycine, 20% methanol (v/v) at 100 V for 1 hour. MP and MP mutant proteins were detected by using affinity-purified rabbit antibodies that were raised against synthetic peptides corresponding to amino acid residues 6 to 22 of MP (N-terminal anti-MP, [42]) and a goat-anti-rabbit igG conjugated to horseradish peroxidase (Santa Cruz Biotechnology). The ECL Plus Western Blotting Detection Reagents kit (Amersham Biosciences) was used for signal detection. Following MP detection the same membrane was stained with Coomassie blue.

## Supporting Information

Figure S1 Complementation of MP-deficient virus in MP-transgenic *N. benthamiana* plants. It was previously shown in *N. tabacum* that MP-transgenic plants complement for MP-deficient virus [44,77]. As shown, the same also applies to *N. benthamiana* plants. MP-transgenic plants complement MP-deficient virus (TMV-ΔM-GFP).
(0.51 MB TIF)

Figure S2 MP enhances the spread of GFP silencing in systemic leaves of homozygous MP-expressing plants. Efficiency of cell-to-cell spread of GFP silencing during 36 h in segments of upper, non-infiltrated leaves that where homozygous for GFP and carried either no MP (-/-; GFP/GFP, top row) or two doses of MP (MP/MP; GFP/GFP, lower row). The first and second panels in each row show the silencing pattern at 8 dpi and 36 h later (10 dpi), respectively. The third panels show overlays of the first two panels. Blue false color represents the silenced area at 8 dpi (as shown in first panels) and red enhanced color indicates the increase of silenced areas after the 36 h incubation period (as shown in second panels). Panels four and five in each row show similar overlays made from different source images. Like in heterozygous plants (Figure 1C), at 10 dpi the area of newly silenced tissue (shown in red artificial color) was considerably greater in the presence of MP than in its absence.
(0.77 MB TIF)

Figure S3 Transiently expressed MP complements for the spread of MP-deficient TMV-ΔM-GFP. Left panel: wild type leaf infiltrated with empty vector (ev) does not complement TMV-ΔM-GFP; right panel: transient expression of MP in an agroinfiltrated wild type leaf complements TMV-ΔM-GFP. Fluorescent rings (examples marked by arrowheads) indicate the locations on the leaf where agrobacteria where injected. Examples of TMV-ΔM-GFP infection sites are marked by arrows.
(0.54 MB TIF)

Figure S4 Subcellular localization of transiently expressed MP:GFP, PS1:GFP, and dMP:GFP in agroinfiltrated leaves. (A) Cortical view of an epidermal cell showing MP:GFP in association with microtubules. (B) Cortical view of an epidermal cell showing diffuse, non-localized, PS1:GFP fluorescence. (C) Cortical view of an epidermal cell showing dMP:GFP in association with microtubules. (D) Central view of a cell indicating the localization of MP:GFP to plasmodesmata. (E) Central view of a PS1:GFP-expressing cell indicating the lack of localization of the protein to plasmodesmata. (F) Central view of a dMP:GFP-expressing cell. dMP:GFP does not target plasmodesmata efficiently. The cell-wall near signals in this figure are dMP:GFP-associated microtubules that are seen in cross section. All scale bars: 10 µm.
(0.52 MB TIF)

Figure S5 GFP mRNA and siRNA levels in cells expressing MP and MP mutants. (A) GFP siRNAs became visible at 5 dpi when mRNA levels were strongly decreased. In the presence of MP, siRNA levels were reduced, whereas they stayed unaffected in tissues expressing either MP mutant TAD5 or MP mutant PS1. miR166 is shown as a loading control. (B) GFP mRNA and siRNA levels were unchanged in tissues expressing CP. miR165 is shown as a loading control.
(0.66 MB TIF)

Figure S6 Spread of MP:GFP from cells transfected with diluted agrobacteria. (A and B) RMS2 expression in tissues infiltrated with agrobacteria harboring both RMS2- and MP:GFP-encoding plasmids. The agrobacteria were undiluted (OD = 0.04) or diluted (OD = 0.001) before infiltration. In tissues infiltrated with non-diluted bacteria, almost every cell becomes transformed and labeled by the presence of cell-autonomous RMS2 protein in the nucleus (A). In contrast, in tissues infiltrated with diluted bacteria only single individual cells become transformed and are surrounded by non-transformed cells, as shown by the absence of RMS2 labeling (B). The images show merged differential interference contrast (DIC) and red fluorescence channel acquisitions. (C) Merge of a green and red fluorescence channel acquisitions showing the spread of MP:GFP (arrowheads) into cells surrounding the transfected RMS2-labeled cell in tissue treated with diluted agrobacteria. Size bars represent 100 µm (A and B) and 50 µm (C).
(0.83 MB TIF)

Figure S7 Time course of infection (A) Infection of wild type (wt) and homozygous MP-transgenic plants (MP^+^) with TMV-126k^m^-GFP. Without an effective silencing suppressor function provided by the replicase, infection sites show viral silencing in the center. Although transgenic MP may slightly facilitate the spread of the virus and thus the enlargement of infection sites, it has no obvious effect on the occurrence of central silencing. Scale bar is for all panels and represents 5 mm. (B) Infection of homozygous MP-transgenic plants (MP^+^) with TMV-126k^m^-ΔM-GFP. Infection sites caused by this MP-deficient virus enlarge with the same efficiency in MP-transgenic plants as the MP-expressing virus TMV-126k^m^-GFP. However, unlike TMV-126k^m^-GFP infection sites, TMV-126k^m^-ΔM-GFP infection sites do not develop central silencing. Thus, virus-encoded MP appears to contribute to the silencing and control of the virus during late stages of infection. Scale bar is for all panels and represents 5 mm.
(0.19 MB TIF)

## References

[1] Tijsterman M, Ketting RF, Plasterk RHA (2002). The genetics of RNA silencing.. Annu Rev Genet.

[2] Hammond SM (2005). Dicing and slicing: the core machinery of the RNA interference pathway.. FEBS Lett.

[3] Meins F, Si-Ammour A, Blevins T (2005). RNA silencing systems and their relevance to plant development.. Annu Rev Cell Dev Biol.

[4] Herr AJ, Baulcombe DC (2004). RNA silencing pathways in plants.. Cold Spring Harb Symp Quant Biol.

[5] Zamore PD, Haley B (2005). Ribo-gnome: the big world of small RNAs.. Science.

[6] Carrington JC (2005). Small RNAs and Arabidopsis. A fast forward look.. Plant Physiol.

[7] Herr AJ (2005). Pathways through the small RNA world of plants.. FEBS Lett.

[8] Brodersen P, Voinnet O (2006). The diversity of RNA silencing pathways in plants.. Trends Genet.

[9] Tuschl T, Zamore PD, Lehmann R, Bartel DP, Sharp PA (1999). Targeted mRNA degradation by double-stranded RNA *in vitro.*. Genes Dev.

[10] Hamilton A, Voinnet O, Chappel L, Baulcombe D (2002). Two classes of short interfering RNA in RNA silencing.. EMBO J.

[11] Hamilton AJ, Baulcombe DC (1999). A species of small antisense RNA in posttranscriptional gene silencing in plants.. Science.

[12] Baumberger N, Baulcombe DC (2005). Arabidopsis ARGONAUTE1 is an RNA Slicer that selectively recruits microRNAs and short interfering RNAs.. Proc Natl Acad Sci U S A.

[13] Rand TA, Petersen S, Du F, Wang X (2005). Argonaute2 cleaves the anti-guide strand of siRNA during RISC activation.. Cell.

[14] Fagard M, Vaucheret H (2000). Systemic silencing signal(s).. Plant Mol Biol.

[15] Mlotshwa S, Voinnet O, Mette MF, Matzke M, Vaucheret H (2002). RNA silencing and the mobile silencing signal.. Plant Cell Supplement.

[16] Voinnet O, Baulcombe DC (1997). Systemic signaling in gene silencing.. Nature.

[17] Voinnet O, Vain P, Angell S, Baulcombe DC (1998). Systemic spread of sequence-specific transgene RNA degradation in plants is initiated by localized introduction of ectopic promoterless DNA.. Cell.

[18] Voinnet O (2005). Non-cell autonomous RNA silencing.. FEBS Lett.

[19] Heinlein M, Oparka K (2005). Systemic RNA silencing.. Plasmodesmata.

[20] Palauqui J-C, Elmayan T, Pollien J-M, Vaucheret H (1997). Systemic acquired silencing: transgene-specific post-transcriptional silencing is transmitted by grafting from silenced stocks to non-silenced scions.. EMBO J.

[21] Himber C, Dunoyer P, Moissiard G, Ritzenthaler C, Voinnet O (2003). Transitivity-dependent and -independent cell-to-cell movement of RNA silencing.. EMBO J.

[22] Palauqui JC, Vaucheret H (1998). Transgenes are dispensable for the RNA degradation step of cosuppression.. Proc Natl Acad Sci U S A.

[23] Garcia-Perez RD, Houdt HV, Depicker A (2004). Spreading of post-transcriptional gene silencing along the target gene promotes systemic silencing.. Plant J.

[24] Schwach F, Vaistij FE, Jones L, Baulcombe DC (2005). An RNA-dependent RNA polymerase prevents meristem invasion by potato virus X and is required for the activity but not the production of a systemic silencing signal.. Plant Physiol.

[25] Voinnet O (2005). Induction and suppression of RNA silencing: insights from viral infections.. Nat Rev Genet.

[26] Voinnet O (2001). RNA silencing as a plant immune system against viruses.. Trends Genet.

[27] Deleris A, Gallego-Bartolome J, Bao J, Kasschau KD, Carrington JC (2006). Hierarchical action and inhibition of plant Dicer-like proteins in antiviral defense.. Science.

[28] Pfeffer S, Dunoyer P, Heim F, Richards KE, Jonard G (2002). P0 of Beet western yellows virus is a suppressor of posttranscriptional gene silencing.. J Virol.

[29] Pruss G, Ge X, Shi XM, Carrington JC, Vance VB (1997). Plant viral synergism: the potyviral genome encodes a broad-range pathogenicity enhancer that transactivates replication of heterologous viruses.. Plant Cell.

[30] Lucas WJ (2006). Plant viral movement proteins: agents for cell-to-cell trafficking of viral genomes.. Virology.

[31] Waigmann E, Lucas W, Citovsky V, Zambryski P (1994). Direct functional assay for tobacco mosaic virus cell-to-cell movement protein and identification of a domain involved in increasing plasmodesmal permeability.. Proc Natl Acad Sci USA.

[32] Deom CM, Oliver MJ, Beachy RN (1987). The 30-kilodalton gene product of tobacco mosaic virus potentiates virus movement.. Science.

[33] Wolf S, Deom CM, Beachy RN, Lucas WJ (1989). Movement protein of tobacco mosaic virus modifies plasmodesmatal size exclusion limit.. Science.

[34] Citovsky V, Knorr D, Schuster G, Zambryski P (1990). The P30 movement protein of tobacco mosaic virus is a single-stranded nucleic acid binding protein.. Cell.

[35] Heinlein M, Epel BL, Padgett HS, Beachy RN (1995). Interaction of tobamovirus movement proteins with the plant cytoskeleton.. Science.

[36] Boyko V, Ferralli J, Ashby J, Schellenbaum P, Heinlein M (2000). Function of microtubules in intercellular transport of plant virus RNA.. Nat Cell Biol.

[37] Boyko V, Hu Q, Seemanpillai M, Ashby J, Heinlein M (2007). Validation of microtubule-associated *Tobacco mosaic virus* RNA movement and involvement of microtubule-aligned particle trafficking.. Plant J.

[38] Heinlein M, Padgett HS, Gens JS, Pickard BG, Casper SJ (1998). Changing patterns of localization of the *Tobacco mosaic virus* movement protein and replicase to the endoplasmic reticulum and microtubules during infection.. Plant Cell.

[39] Más P, Beachy RN (1999). Replication of tobacco mosaic virus on endoplasmic reticulum and role of the cytoskeleton and virus movement in intracellular distribution of viral RNA.. J Cell Biol.

[40] Ruiz MT, Voinnet O, Baulcombe DC (1998). Initiation and maintenance of virus-induced gene silencing.. Plant Cell.

[41] Giesman-Cookmeyer D, Silver S, Vaewhongs AA, Lommel SA, Deom CM (1995). Tobamovirus and dianthovirus movement proteins are functionally homologous.. Virology.

[42] Boyko V, van der Laak J, Ferralli J, Suslova E, Kwon M-O (2000). Cellular targets of functional and dysfunctional mutants of tobacco mosaic virus movement protein fused to GFP.. J Virol.

[43] Kotlizky G, Katz A, van der Laak J, Boyko V, Lapidot M (2001). A dysfunctional movement protein of *Tobacco mosaic virus* interferes with targeting of wild type movement protein to microtubules.. Mol Plant Microbe Interact.

[44] Lapidot M, Gafny R, Ding B, Wolf S, Lucas WJ (1993). A dysfunctional movement protein of tobacco mosaic virus that partially modifies the plasmodesmata and limits spread in transgenic plants.. Plant J.

[45] Kahn TW, Lapidot M, Heinlein M, Reichel C, Cooper B (1998). Domains of the TMV movement protein involved in subcellular localization.. Plant J.

[46] Deom CM, He XZ (1997). Second-site reversion of a dysfunctional mutation in a conserved region of the tobacco mosaic virus movement protein.. Virology.

[47] Boyko V, Ashby JA, Suslova E, Ferralli J, Sterthaus O (2002). Intramolecular complementing mutations in *Tobacco mosaic virus* movement protein confirm a role for microtubule association in viral RNA transport.. J Virol.

[48] Dunoyer P, Himber C, Voinnet O (2005). DICER-LIKE 4 is required for RNA interference and produces the 21-nucleotide small interfering RNA component of the plant cell-to-cell silencing signal.. Nat Genet.

[49] Lakatos L, Csorba T, Pantaleo V, Chapman EJ, Carrington JC (2006). Small RNA binding is a common strategy to suppress RNA silencing by several viral suppressors.. EMBO J.

[50] Itaya A, Hickman H, Bao Y, Nelson R, Ding B (1997). Cell-to-cell trafficking of cucumber mosaic virus movement protein:green fluorescent protein fusion produced by biolistic gene bombardment in tobacco.. Plant J.

[51] Kubota K, Tsuda S, Tamai A, Meshi T (2003). *Tomato mosaic virus* replication protein suppresses virus-targeted posttranscriptional gene silencing.. J Virol.

[52] Vogler H, Akbergenov R, Dang V, Fasler M, Shivaprasad PV (2007). Small RNA modification associated with suppression of RNA silencing by tobamovirus replicase protein.. J Virol.

[53] Molnar A, Csorba T, Lakatos L, Varallyay E, Lacomme C (2005). Plant virus-derived small interfering RNAs originate predominantly from highly structured single-stranded viral RNAs.. J Virol.

[54] Kurihara Y, Inaba N, Kutsuna N, Takeda A, Tagami Y (2007). Binding of tobamovirus replication protein with small RNA duplexes.. J Gen Virol.

[55] Reichel C, Más P, Beachy RN (1999). The role of the ER and cytoskeleton in plant viral trafficking.. Trends Plant Sci.

[56] Simon AE, Roossinck MJ, Havelda Z (2004). Plant virus satellite and defective interfering RNAs: New paradigms for a new century.. Annu Rev Phytopathology.

[57] Pfeffer S, Zavolan M, Grasser FA, Chien M, Russo JJ (2004). Identification of virus-encoded microRNAs.. Science.

[58] Sullivan CS, Grundhoff AT, Tevethia S, Pipas JM, Ganem D (2005). SV40-encoded microRNAs regulate viral gene expression and reduce susceptibility to cytotoxic T cells.. Nature.

[59] Ratcliff F, Harrison BD, Baulcombe DC (1997). A similarity between viral defense and gene silencing in plants.. Science.

[60] Oparka KJ, Prior DAM, Santa Cruz S, Padgett HS, Beachy RN (1997). Gating of epidermal plasmodesmata is restricted to the leading edge of expanding infection sites of tobacco mosaic virus.. Plant J.

[61] Sacristan S, Malpica JM, Fraile A, Garcia-Arenal F (2003). Estimation of population bottlenecks during systemic movement of tobacco mosaic virus in tobacco plants.. J Virol.

[62] Szécsi J, Ding XS, Lim CO, Bendahmane M, Cho MJ (1999). Development of tobacco mosaic virus infection sites in *Nicothiana benthamiana.*. Mol Plant Microbe Interact.

[63] Lehto K, Bubrick P, Dawson WO (1990). Time course of TMV 30k protein accumulation in intact leaves.. Virology.

[64] Heinlein M (2002). The spread of *Tobacco mosaic virus* infection: insights into the cellular mechanism of RNA transport.. Cell Mol Life Sci.

[65] Foster TM, Lough TJ, Emerson SJ, Lee RH, Bowman JL (2002). A surveillance system regulates selective entry of RNA into the shoot apex.. Plant Cell.

[66] Boyko V, Ferralli J, Heinlein M (2000). Cell-to-cell movement of TMV RNA is temperature-dependent and corresponds to the association of movement protein with microtubules.. Plant J.

[67] Citovsky V, Wong ML, Shaw AL, Venkataram Prasad BV, Zambryski P (1992). Visualization and characterization of tobacco mosaic virus movement protein binding to single-stranded nucleic acids.. Plant Cell.

[68] Kobayashi K, Zambryski P (2007). RNA silencing and its cell-to-cell spread during Arabidopsis embryogenesis.. Plant J.

[69] Yoo BC, Kragler F, Varkonyi-Gasic E, Haywood V, Archer-Evans S (2004). A systemic small RNA signaling system in plants.. Plant Cell.

[70] Zhang X, Yuan YR, Pei Y, Lin SS, Tuschl T (2006). Cucumber mosaic virus-encoded 2b suppressor inhibits Arabidopsis Argonaute1 cleavage activity to counter plant defense.. Genes Dev.

[71] Gafny R, Lapidot M, Berna A, Holt CA, Deom CM (1992). Effects of terminal deletion mutations on function of the movement protein of tobacco mosaic virus.. Virology.

[72] Holt CA, Beachy RN (1991). In vivo complementation of infectious transcripts from mutant tobacco mosaic virus cDNAs in transgenic plants.. Virology.

[73] Hellens RP, Edwards EA, Leyland NR, Bean S, Mullineaux PM (2000). pGreen: a versatile and flexible binary Ti vector for Agrobacterium-mediated plant transformation.. Plant Mol Biol.

[74] Ashby J, Boutant E, Seemanpillai M, Groner A, Sambade A (2006). *Tobacco mosaic virus* movement protein functions as a structural microtubule-associated protein.. J Virol.

[75] Fouts D, True H, Celander D (1997). Functional recognition of fragmented operator sites by R17/MS2 coat protein, a translational repressor.. Nucl Acid Res.

[76] Voinnet O, Rivas S, Mestre P, Baulcombe D (2003). An enhanced transient expression system in plants based on suppression of gene silencing by the p19 protein of tomato bushy stunt virus.. Plant J.

