# Supplementary figures and images for: Tobacco mosaic virus Movement Protein Enhances the Spread of RNA Silencing
Source: PLoS Pathog. 2008 Apr 4;4(4):e1000038. doi: 10.1371/journal.ppat.1000038 (PMC2270343; doi:10.1371/journal.ppat.1000038)

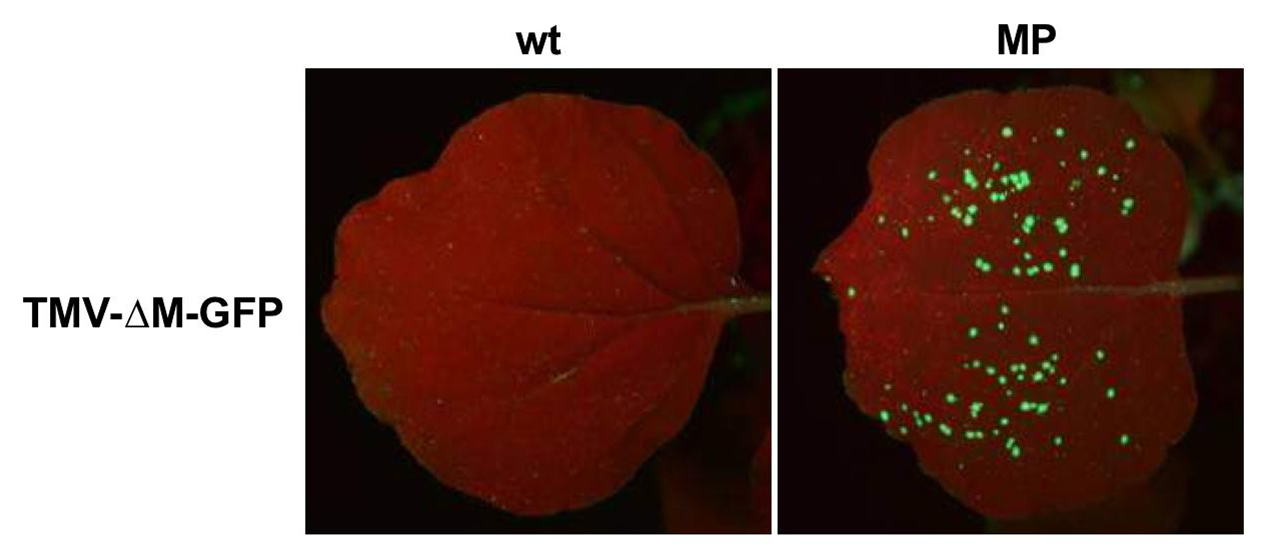

Supplement: Figure S1 — Complementation of MP-deficient virus in MP-transgenic N. benthamiana plants. It was previously shown in N. tabacum that MP-transgenic plants complement for MP-deficient virus [44,77]. As shown, the same also applies to N. benthamiana plants. MP-transgenic plants complement MP-deficient virus (TMV-ΔM-GFP). (0.51 MB TIF) [file ppat.1000038.s001.tif]

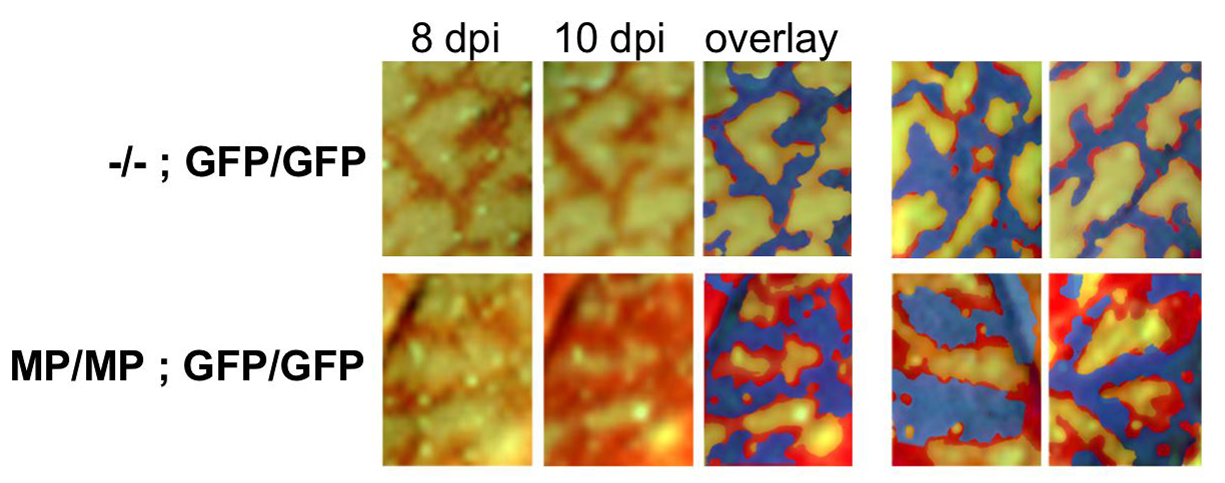

Supplement: Figure S2 — MP enhances the spread of GFP silencing in systemic leaves of homozygous MP-expressing plants. Efficiency of cell-to-cell spread of GFP silencing during 36 h in segments of upper, non-infiltrated leaves that where homozygous for GFP and carried either no MP (-/-; GFP/GFP, top row) or two doses of MP (MP/MP; GFP/GFP, lower row). The first and second panels in each row show the silencing pattern at 8 dpi and 36 h later (10 dpi), respectively. The third panels show overlays of the first two panels. Blue false color represents the silenced area at 8 dpi (as shown in first panels) and red enhanced color indicates the increase of silenced areas after the 36 h incubation period (as shown in second panels). Panels four and five in each row show similar overlays made from different source images. Like in heterozygous plants (Figure 1C), at 10 dpi the area of newly silenced tissue (shown in red artificial color) was considerably greater in the presence of MP than in its absence. (0.77 MB TIF) [file ppat.1000038.s002.tif]

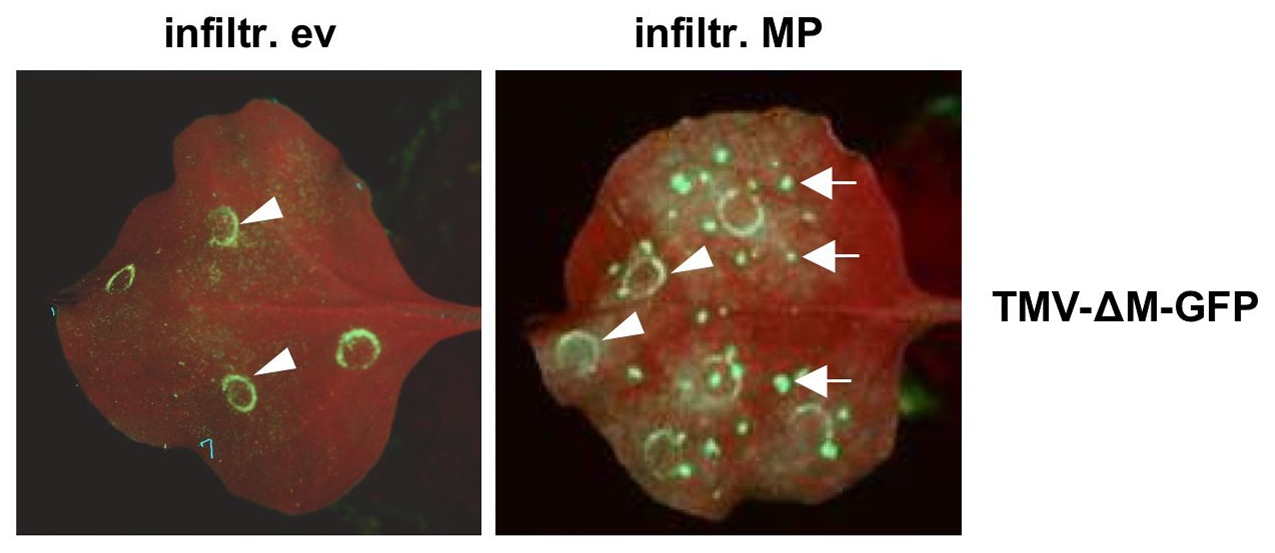

Supplement: Figure S3 — Transiently expressed MP complements for the spread of MP-deficient TMV-ΔM-GFP. Left panel: wild type leaf infiltrated with empty vector (ev) does not complement TMV-ΔM-GFP; right panel: transient expression of MP in an agroinfiltrated wild type leaf complements TMV-ΔM-GFP. Fluorescent rings (examples marked by arrowheads) indicate the locations on the leaf where agrobacteria where injected. Examples of TMV-ΔM-GFP infection sites are marked by arrows. (0.54 MB TIF) [file ppat.1000038.s003.tif]

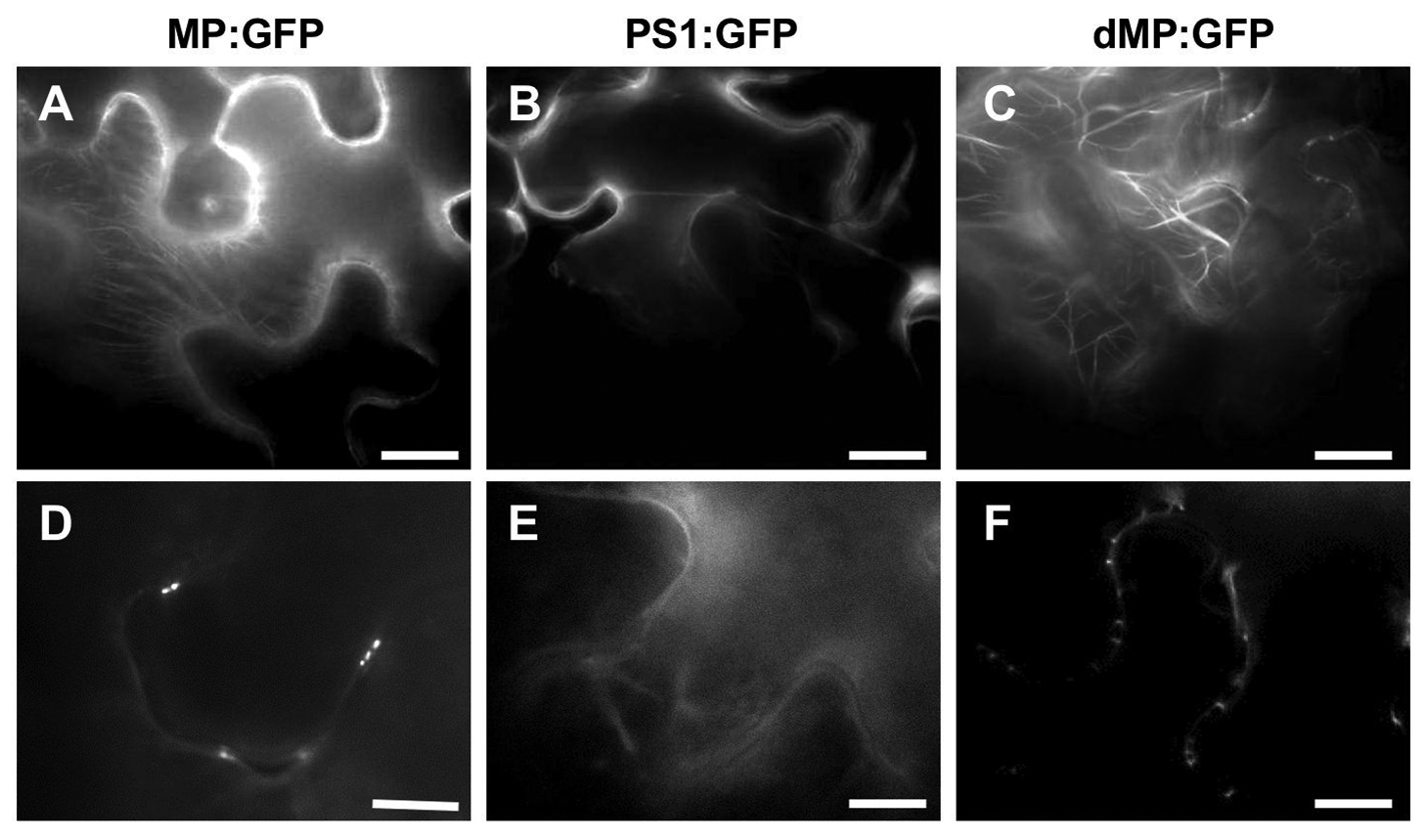

Supplement: Figure S4 — Subcellular localization of transiently expressed MP:GFP, PS1:GFP, and dMP:GFP in agroinfiltrated leaves. (A) Cortical view of an epidermal cell showing MP:GFP in association with microtubules. (B) Cortical view of an epidermal cell showing diffuse, non-localized, PS1:GFP fluorescence. (C) Cortical view of an epidermal cell showing dMP:GFP in association with microtubules. (D) Central view of a cell indicating the localization of MP:GFP to plasmodesmata. (E) Central view of a PS1:GFP-expressing cell indicating the lack of localization of the protein to plasmodesmata. (F) Central view of a dMP:GFP-expressing cell. dMP:GFP does not target plasmodesmata efficiently. The cell-wall near signals in this figure are dMP:GFP-associated microtubules that are seen in cross section. All scale bars: 10 µm. (0.52 MB TIF) [file ppat.1000038.s004.tif]

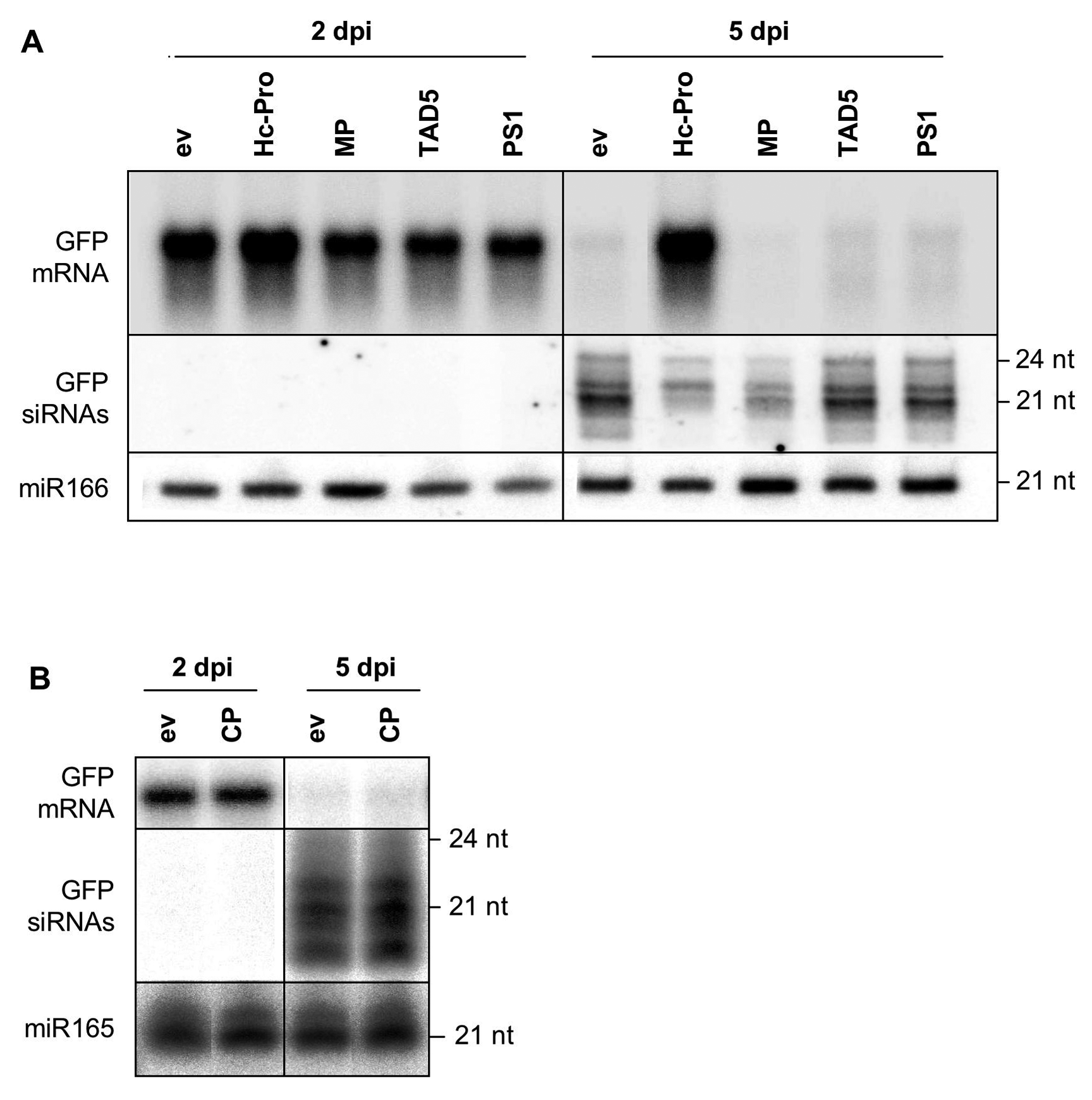

Supplement: Figure S5 — GFP mRNA and siRNA levels in cells expressing MP and MP mutants. (A) GFP siRNAs became visible at 5 dpi when mRNA levels were strongly decreased. In the presence of MP, siRNA levels were reduced, whereas they stayed unaffected in tissues expressing either MP mutant TAD5 or MP mutant PS1. miR166 is shown as a loading control. (B) GFP mRNA and siRNA levels were unchanged in tissues expressing CP. miR165 is shown as a loading control. (0.66 MB TIF) [file ppat.1000038.s005.tif]

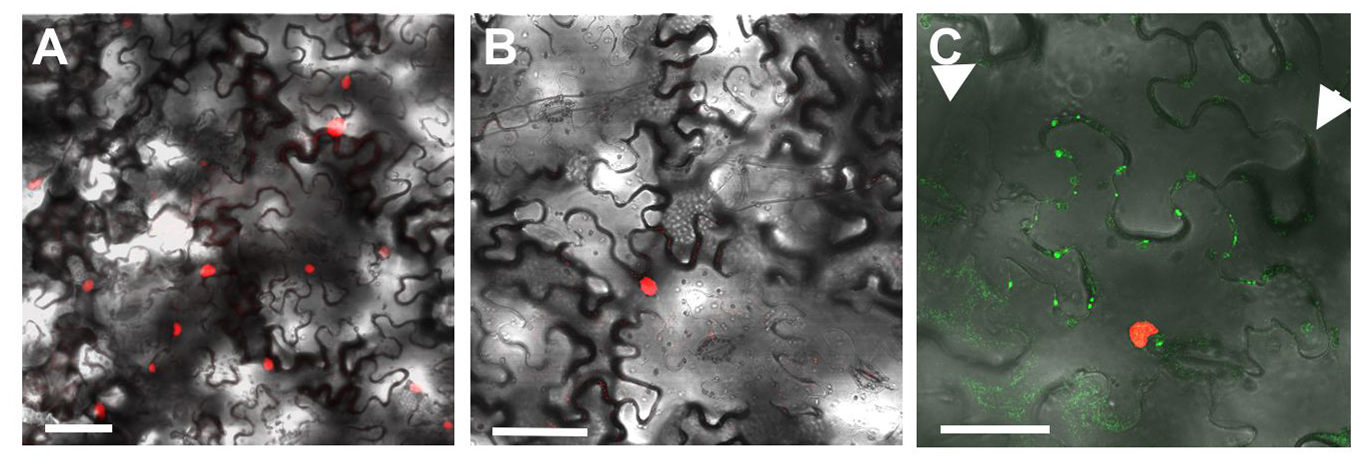

Supplement: Figure S6 — Spread of MP:GFP from cells transfected with diluted agrobacteria. (A and B) RMS2 expression in tissues infiltrated with agrobacteria harboring both RMS2- and MP:GFP-encoding plasmids. The agrobacteria were undiluted (OD = 0.04) or diluted (OD = 0.001) before infiltration. In tissues infiltrated with non-diluted bacteria, almost every cell becomes transformed and labeled by the presence of cell-autonomous RMS2 protein in the nucleus (A). In contrast, in tissues infiltrated with diluted bacteria only single individual cells become transformed and are surrounded by non-transformed cells, as shown by the absence of RMS2 labeling (B). The images show merged differential interference contrast (DIC) and red fluorescence channel acquisitions. (C) Merge of a green and red fluorescence channel acquisitions showing the spread of MP:GFP (arrowheads) into cells surrounding the transfected RMS2-labeled cell in tissue treated with diluted agrobacteria. Size bars represent 100 µm (A and B) and 50 µm (C). (0.83 MB TIF) [file ppat.1000038.s006.tif]

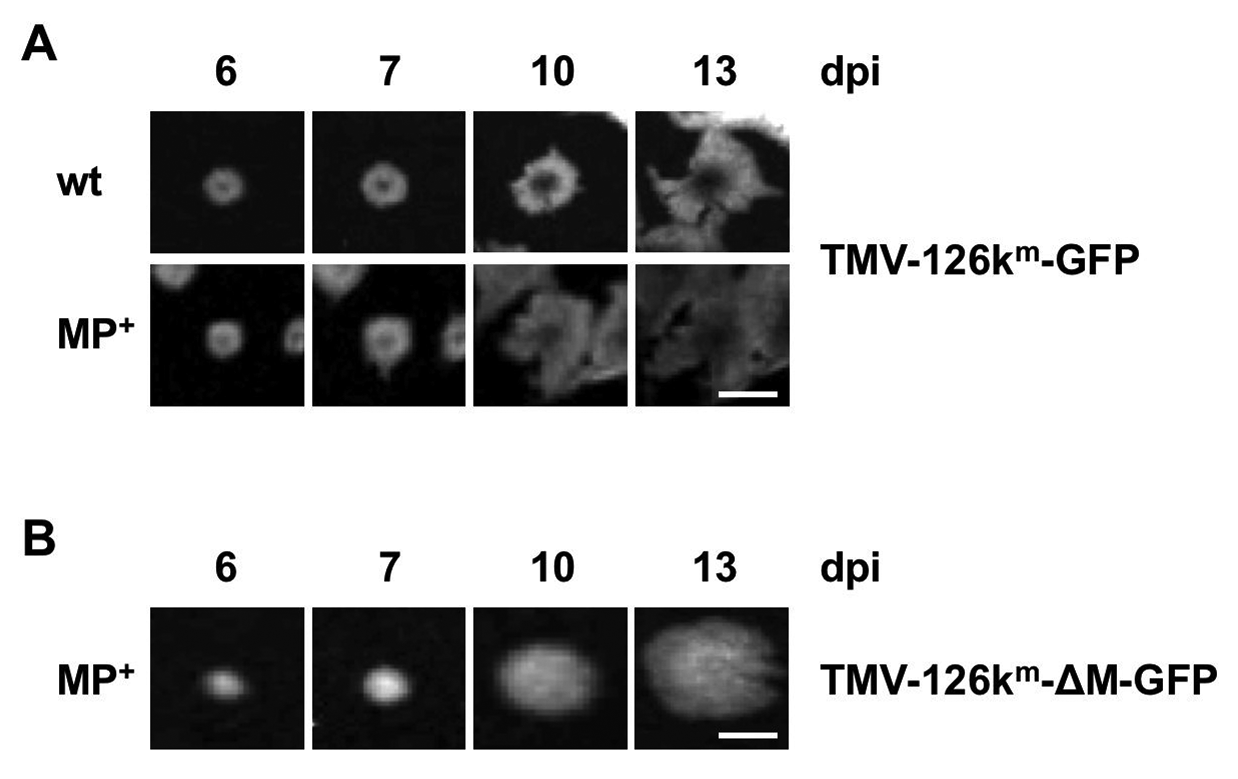

Supplement: Figure S7 — Time course of infection (A) Infection of wild type (wt) and homozygous MP-transgenic plants (MP+) with TMV-126km-GFP. Without an effective silencing suppressor function provided by the replicase, infection sites show viral silencing in the center. Although transgenic MP may slightly facilitate the spread of the virus and thus the enlargement of infection sites, it has no obvious effect on the occurrence of central silencing. Scale bar is for all panels and represents 5 mm. (B) Infection of homozygous MP-transgenic plants (MP+) with TMV-126km-ΔM-GFP. Infection sites caused by this MP-deficient virus enlarge with the same efficiency in MP-transgenic plants as the MP-expressing virus TMV-126km-GFP. However, unlike TMV-126km-GFP infection sites, TMV-126km-ΔM-GFP infection sites do not develop central silencing. Thus, virus-encoded MP appears to contribute to the silencing and control of the virus during late stages of infection. Scale bar is for all panels and represents 5 mm. (0.19 MB TIF) [file ppat.1000038.s007.tif]
